# Magnesium sulfate for fetal neuroprotection in preterm pregnancy: a meta-analysis of randomized controlled trials

**DOI:** 10.1186/s12884-024-06703-9

**Published:** 2024-08-01

**Authors:** Kyana Jafarabady, Arman Shafiee, Nasim Eshraghi, Seyyed Amirhossein Salehi, Ida Mohammadi, Shahryar Rajai, Zahra Zareian, Fatemeh Movahed, Mahmood Bakhtiyari

**Affiliations:** 1 Student Research Committee, School of Medicine, Alborz University of Medical Sciences, Karaj, Iran; 2 Department of Psychiatry and Mental Health, Alborz University of Medical Sciences, Karaj, Iran; 3 Student Research Committee, School of Medicine, Tehran University of Medical Sciences, Tehran, Iran; 4 Student Research Committee, School of Medicine, Shahid Beheshti University of Medical Sciences, Tehran, Iran; 5 Department of Obstetrics and Gynaecology, Jahrom School oh medical Science, Jahrom, Iran; 6 Department of Obstetrics and Gynaecology, Tehran University of Medical Sciences, Tehran, Iran; 7 Non-Communicable Diseases Research Centre, Alborz University of Medical Sciences, Karaj, Iran

**Keywords:** Magnesium sulfate, Neuroprotection, Preterm pregnancy

## Abstract

**Background:**

Intravenous administration of magnesium sulfate (MgSO4) to expectant individuals before childbirth, has been evaluated to reduce the likelihood of mortality and occurrence cerebral palsy in their offspring. Therefore, this systematic review and meta-analysis conducted to determine if were the prophylactic use of magnesium sulfate in women at risk for preterm delivery leads to decrease in the incidence of death or cerebral palsy.

**Methods:**

A comprehensive search of electronic databases was done to identify relevant studies. Selection of eligible studies was based on predetermined inclusion criteria. Data extraction was performed, and the methodological quality of the selected studies was assessed using appropriate evaluative tools. A meta-analysis was carried out to estimate the overall effect of intravenous administration of magnesium sulfate on the incidence of death or cerebral palsy.

**Results:**

A total of 7 studies met the inclusion criteria and were included in the final analysis. No significant publication bias was observed. The risk of fetal neurological impairment was significantly lower in the MgSO4 group compared to the control group relative risk (RR = 0.70, 95% CI: 0.56 to 0.87; I20%). However, neonatal mortality was not significantly associated with MgSO4 injection. (RR = 1.03, 95% CI: 0.88 to 1.21; I2 = 42%). Subgroup analysis was done based on the bolus dosage of MgSO4 and the duration of the trial follow-up. revealing a non-significant differences between-group.

**Conclusion:**

This study demonstrated that MgSO4 administration can improve fetal neurological impairment and cerebral palsy but is not linked to reducing mortality. Further studies are necessary to strengthen the evidence and clarify the underlying mechanisms.

**Supplementary Information:**

The online version contains supplementary material available at 10.1186/s12884-024-06703-9.

## Introduction

The prevalence of preterm birth ranges from approximately 5–18% among all pregnancies, with an observed increasing trend in recent years [1, 2]. Additionally, a significant number of deaths in children under the age of 5 are attributed to premature birth and its associated complications [3]. Identifying strategies to mitigate the risks associated with preterm births is crucial, as they are known to be linked to a heightened risk of neurodevelopmental challenges. This recognition underscores the importance of implementing effective measures to minimize the potential long-term impacts on the neurodevelopment of preterm infants [4].

A wide range of interventions have been extensively studied in the quest to prevent preterm birth and mitigate the neurological consequences for preterm infants. Among these interventions, magnesium sulfate (MgSO_4_) has emerged as a particularly promising agent for fetal neuroprotection [5]. Although the exact mechanism by which magnesium sulfate reduces the risk of brain injury remains unclear, several potential mechanisms have been proposed. These include reducing cerebral inflammation and hemorrhage, protecting against excitotoxicity, improving vasodilation and blood supply to the brain, and promoting neuronal maturation and survival [6, 7].

While some previous randomized clinical trials and meta-analyses have suggested that antenatal exposure to MgSO_4_ could reduce the risk of subsequent cerebral palsy among neonates [8–10], a recent trial called the MAGENTA trial, published in JAMA [11], found that MgSO_4_ administration during weeks 30–34 of gestation did not increase the likelihood of survival without cerebral palsy in children. As a result, there are still unanswered questions regarding the optimal timing of MgSO4 administration and the appropriate dosage. Additionally, it is crucial to conduct comprehensive investigations into the potential complications associated with the use of MgSO_4_ [12].

In this systematic review and meta-analysis, we aimed to evaluate the impact of administering MgSO_4_ to women at risk of preterm birth with gestational age on the occurrence of neurological impairments and mortality rates, by analyzing data from all published randomized controlled trials (RCTs).

## Method

This investigation was conducted following the guidelines outlined in the Preferred Reporting Items for Systematic Reviews and Meta-Analyses (PRISMA) statement [8]. Our study protocol is registered at PROSPERO under the number CRD42023455978.

### Search strategy

We conducted a systematic search across different databases, including Medline/PubMed, Cochrane database (CENTRAL), and Clinicaltrials.gov, to identify relevant studies. The search was carried out until November 2023. We utilized specific search terms to evaluate the association between the neuroprotective effect of antenatal MgSO_4_ administration in preterm fetuses (detailed in Supplementary Table 1).

### Eligibility criteria

In conducting this comprehensive meta-analysis, we systematically incorporated randomized clinical trials (RCTs) published in the English language that specifically investigated the effects of intravenous administration of MgSO_4_ to pregnant women at risk of near preterm delivery, in comparison to a placebo. Only studies that assessed neurological impairments in neonates for one year or longer and reported neonatal mortality rates were eligible for inclusion in this meta-analysis. Non-randomized trials, observational studies, review articles, and letters were excluded.

### Data extraction

Two experienced reviewers independently extracted data from the included studies based on the predefined screening criteria. Any discrepancies between the reviewers’ assessments were resolved through consultation with another expert author to ensure accuracy and consistency. Both abstracts and titles were initially screened, followed by a thorough evaluation of full-text articles. The extracted information encompassed various aspects of the included studies, including the year of publication, first author’s name, sample size, study design, dosage of intravenous MgSO_4_, gestational age, neurodevelopment outcomes (specifically cerebral palsy), and neonatal mortality.

### Risk of bias assessment

In assessing the included trials, we employed the Cochrane Risk of Bias 2 tool to evaluate the risk of bias in each study. By utilizing this standardized approach, we aimed to ensure a comprehensive evaluation of the methodological quality of each trial. Furthermore, we determined the evidence certainty for the main endpoint using the GRADE.

### Data analysis

We employed a random effect model due to variations in study methodologies. Our analyses presented risk ratios with a 95% confidence interval (CI) for categorical outcomes and standardized mean differences with a 95% CI for continuous outcomes. To illustrate heterogeneity, we reported the I2 value. All statistical analyses were performed using R.

## Result

**Fig. 1:**
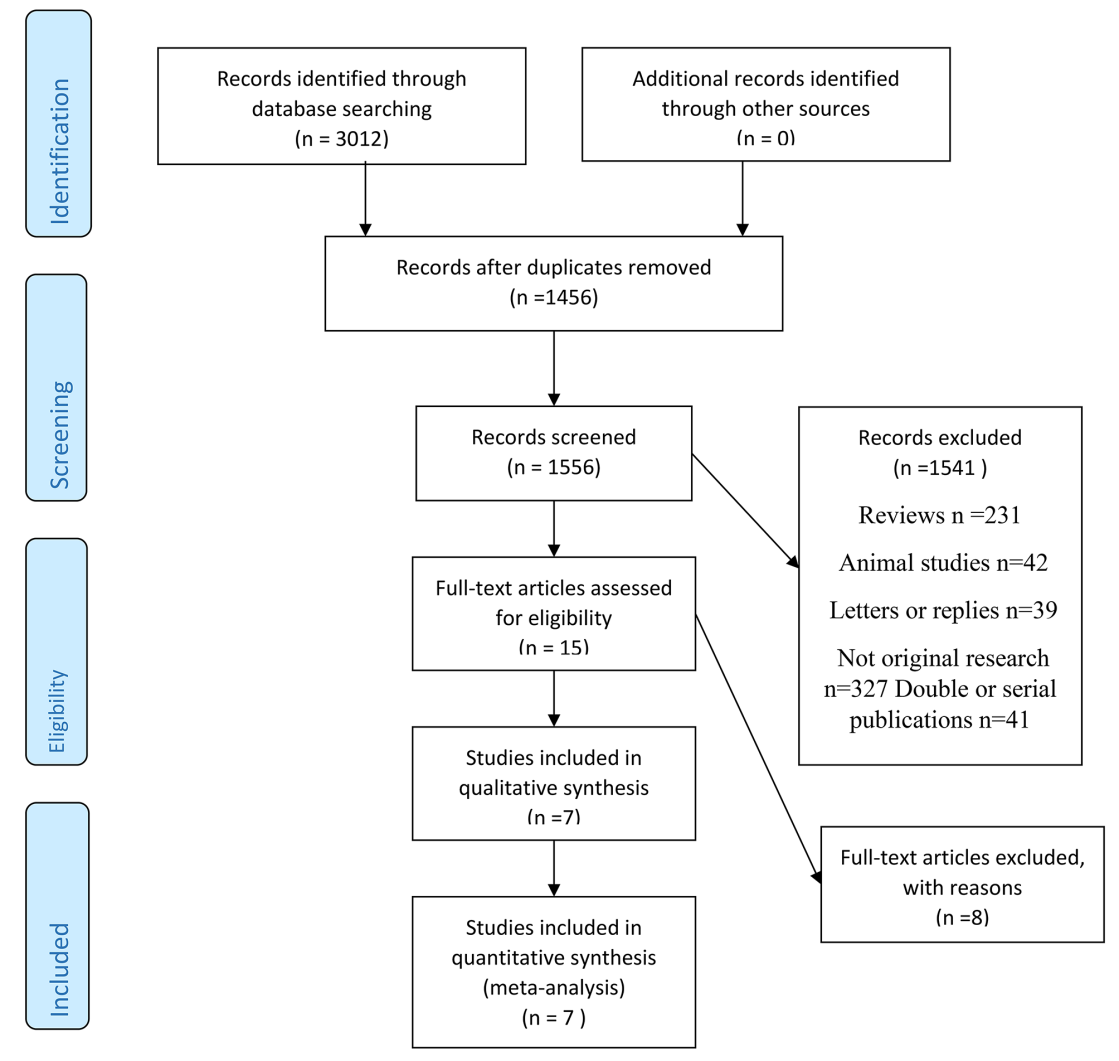
PRISMA 2020 flow diagram for new systematic reviews which included searches of databases and registers only

Initially, a total of 3,012 studies were identified through the initial search. After removing duplicate studies, 1,456 unique studies remained. Subsequently, the abstracts of these studies were screened, resulting in the inclusion of 7 full-text articles for further analysis. Figure 1, depicted in the PRISMA flowchart, provides a visual representation of the selection process.

**Fig. 2:**
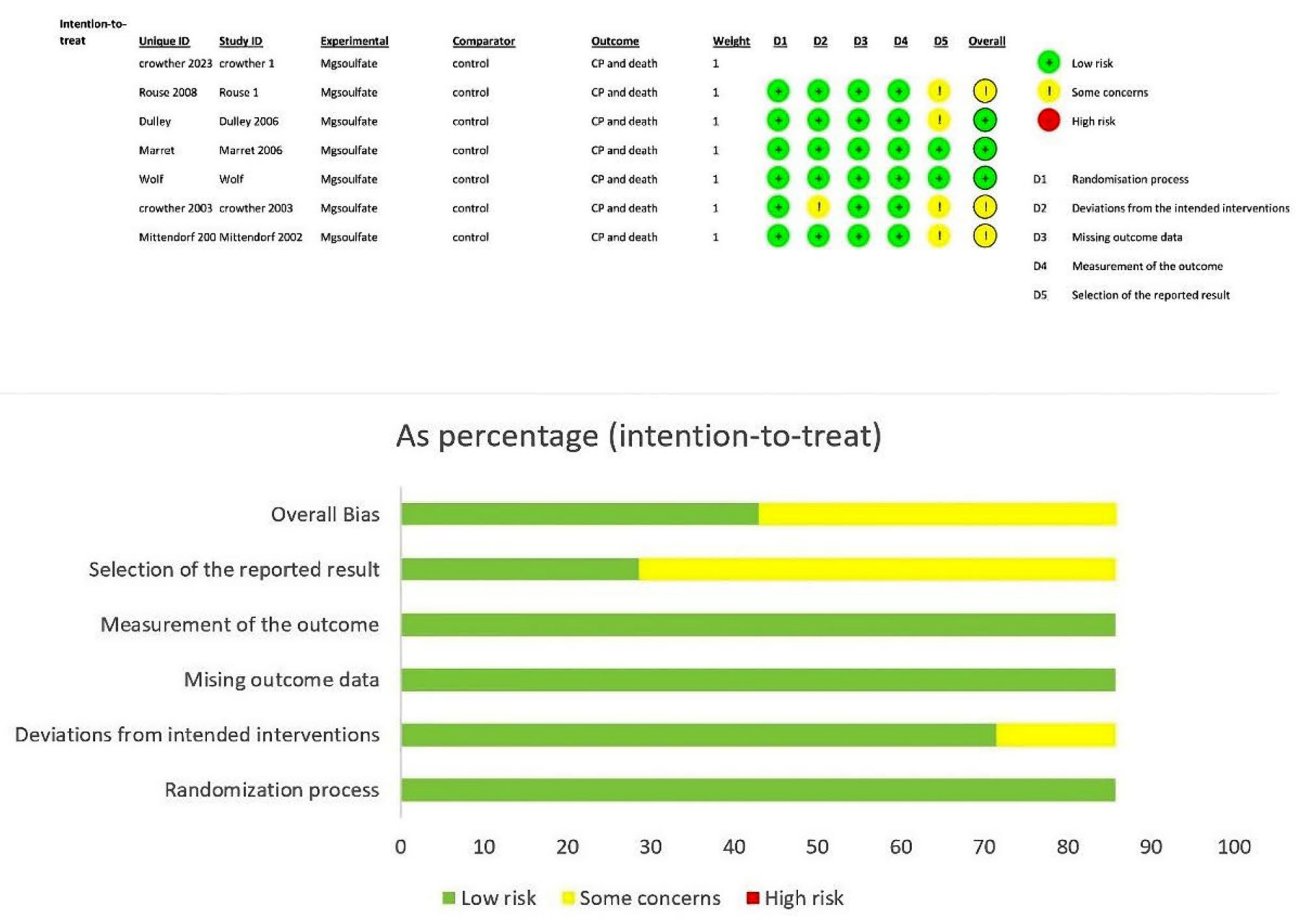
Risk of bias assessment for each included study

The analysis comprised a total of 7 trials, involving a combined participant population of 8,171 individuals. Additional details regarding each trial can be found in Table 1; Fig. 2. All of the studies focused on examining fetal death, as well as the mortality and outcomes of CP in preterm neonates. Follow-up assessments were conducted between 12 and 24 months after birth to evaluate these outcomes. Although all of the women included in the studies had preterm fetuses, the gestational age of the pregnant women varied across the different trials. In most of the studies, a bolus dose of 4 g of MgSO_4_ was administered intravenously. However, it is worth noting that Rouse et al. utilized a dosage of 6 g of MgSO_4_, while Wolf et al. used 5 g of MgSO_4_ [9, 10].

**Table 1:** Baseline characteristics of the patients and trials included

Author	Year	Country	Population (Gestational age)	Exclusion criteria	Duration	MgSO_4_ dosage	Follow up	Quality
Mittendorf	2002	USA	149 (25–33)	Infection, Preeclampsia, plurality > twins	4 years	Bolus dose (g^*^): 4 Maintenance dose (g/h^*^): 0 or 2–3	18 months	Good
Crowther	2003	Australia, New Zealand	1062 (< 30)	Maternal contraindications, second stage of labor	NR	Bolus dose (g): 4 Maintenance dose (g/h): 2	24 months	Good
Duley	2006	33 countries	10,141 (no limitations)	Maternal contraindications	3years	Bolus dose (g): 4 Maintenance dose (g/h): 1	18 months	Good
Marret	2006	France	573 (< 33)	fetal anomalies, threatened fetus, pregnancy associated vascular disease, maternal contraindications, plurality > triplets	NR	Bolus dose (g): 4 Maintenance dose (g/h): 0	24 months	Fair
Rouse	2008	USA	2241 (24–31)	Preeclampsia, cervical dilation more than 8 cm, hypertension, PPROM less than 22 gestation weeks, fetal anomalies, plurality > twins	NR	Bolus dose (g): 6 Maintenance dose (g/h):2	24 months	Good
Wolf	2019	Denmark	560 (24–31)	Major fetal anomalies, maternal contraindications, MgSO_4_ administered for other reasons, plurality > twins	12 months	Bolus dose (g): 5 Maintenance dose (g/h): 1	24 months	Good
Crowther	2023	Australia & New Zealand	1433 (30–34)	MgSO_4_ contraindication (respiratory depression, hypotension, absent patellar reflexes, kidney failure, or myasthenia gravis) or MgSO_4_ therapy was essential for preeclampsia.	6 years	Bolus dose (g): 4 Maintenance dose (g/h): NR	24 months	Good

NR = Not reported/ g = gram/ g/h = gram per hour

The analysis indicates that the risk of fetal neurological impairment was significantly lower in the MgSO4 group compared to the control group. The pooled relative risk (RR) was 0.70 (95% CI: 0.56 to 0.87; I2 = 0%), showing a statistically significant reduction. There was no observed heterogeneity among the studies (I2 = 0), indicating consistent results across the trials.

**Fig. 3:**
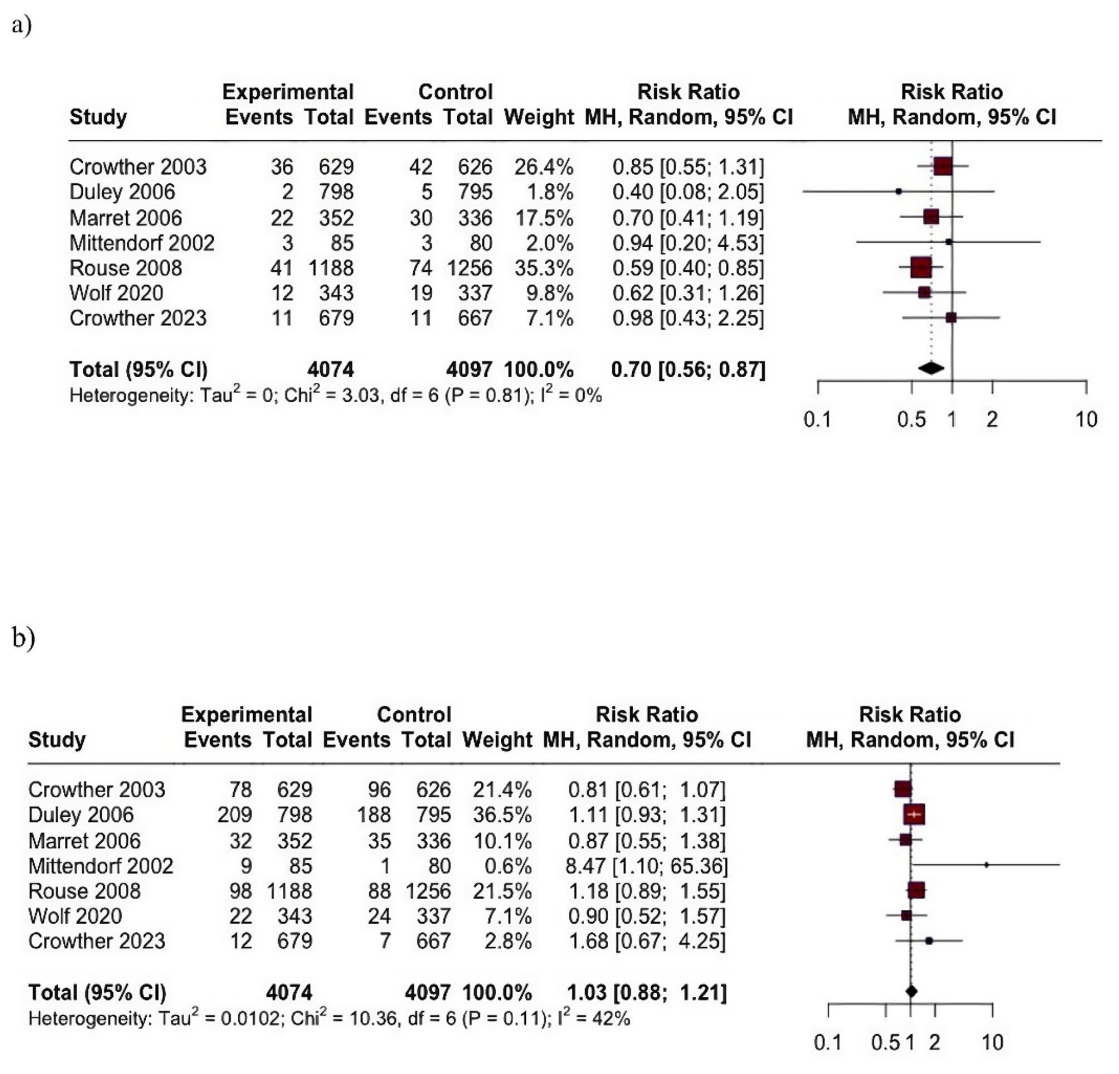
Results of meta-analysis (**a**) fetal neurological impairment; and (**b**) mortality95% CI: 95% confident interval

In term of neonatal mortality, the combined results of the included RCTs showed no significant difference. The RR was 1.03 (95% CI: 0.88 to 1.21; I2 = 42%) (Fig. 3). The I2 value of 42% suggests moderate heterogeneity among the studies, indicating some variability in the results across the trials, although the difference was not statistically significant.

Subgroup analyses were conducted based on the bolus dosage of MgSO_4_ and the duration of the trial follow-up. These analyses indicated that there were no significant differences between the groups in terms of mortality and cerebral palsy (CP). This finding confirmed that the results were not affected by variations in the duration of follow-up or the initial dosage of MgSO_4_ (See supplementary Fig. 1).

## Discussion

The main conclusion of this systematic review and meta-analysis is that the administration of MgSO_4_ reduces the risk of developing CP without impacting the mortality rate. Additionally, our findings indicate that neither the initial dosage of Magnesium sulfate nor the duration of the trial follow-up affects its effectiveness in preventing CP or mortality. Our analysis encompassed seven trials involving a total of 8,171 participants. Furthermore, we conducted subgroup analyses based on MgSO_4_ dosage and trial follow-up duration, and the results in these subgroups were largely consistent with the overall findings.

Preterm pregnancy has been recognized as a risk factor for neurodevelopmental impairments [11]. Among these impairments, visual impairment is more prevalent in preterm infants, particularly among those born extremely prematurely [12]. Additionally, compared to full-term infants, preterm infants are more susceptible to experiencing social-emotional and academic performance difficulties [13]. It is worth noting that these deficits are more commonly observed than severe neurological conditions such as CP, seizures, and significant visual or hearing disorders [14]. However, considering the long-term complications, CP stands out as the most significant and challenging neurological impairment associated with preterm birth.

Magnesium sulfate, which has been used as a tocolytic agent since its first report in 1977, has been considered a potential preventive measure against cerebral palsy and other neurodevelopmental impairments when administered antenatally in cases of anticipated preterm labor [15, 16]. However, the MAGENTA Randomized Clinical Trial, conducted by Crowther, C. A., et al., concluded that the administration of MgSO_4_ for neuroprotection did not improve the likelihood of a child living a cerebral palsy-free life [17]. As a result, we investigated other recently published clinical trials to determine if their findings were consistent with the aforementioned research.

To date, there have been several systematic reviews and meta-analyses conducted on the subject of MgSO_4_ for neuroprotection in preterm births. The systematic review with meta-analysis and trial sequential analysis by Wolf, H. T., et al. included six studies from 2002 to 2019, consisting of a total of 5,917 women. Their findings indicated that MgSO_4_ treatment can serve as an intermediary measure for neuroprotection in pregnant women at imminent risk of preterm delivery [18].

In another systematic review and meta-analysis conducted by Conde-Agudelo, A. and R. Romero, which included a total of six trials, it was deduced that administering MgSO_4_ in women at high risk of preterm delivery before the gestational age of 34 weeks reduces the likelihood of cerebral palsy in their offspring. The prevalence of cerebral palsy among women who did not receive magnesium sulfate as neuroprotection was 5.6%, while the same prevalence among those treated with MgSO_4_ was slightly lower at 3.9% [16].

Furthermore, in the meta-analysis by Crowther, C. A., et al., the efficacy of MgSO_4_ for future CP prevention was investigated. The study included a total of five clinical trials. Based on the results, it was claimed that the administration of MgSO_4_ is highly advisable in women with a heightened possibility of preterm labor. Moreover, this treatment was found to reduce the risk of fatal or infant demise. The observed impacts of this medication were reported to be regardless of the etiology of preterm birth [19]. The findings of these articles align with our research results. The notable contribution of our study is the incorporation of the MAGENTA clinical trial. The outcomes from the MAGENTA research demonstrated a lack of significant difference between the placebo group and the MgSO_4_ group concerning the prevention of cerebral palsy over two years [17].

There are ongoing controversies regarding the optimal cut-off of gestational age and dosage of MgSO_4_ for neuroprotection in preterm fetuses. Various guidelines provide differing recommendations on this matter. Some guidelines suggest considering the use of magnesium sulfate until the end of 32 weeks of gestation, while others recommend its use until 33 weeks and 6 days of gestation [20–22]. It is important to note that the administration of magnesium sulfate for neuroprotection can be associated with maternal and fetal complications. However, previous studies have reported that these side effects do not have a significant impact on maternal and neonatal outcomes [23, 24].

Based on the results of this study and prior systematic reviews and meta-analyses, it can be concluded that the use of MgSO_4_ for neuroprotection is still recommended in pregnancies at risk of preterm birth. However, it is important to consider the conflicting results of the MAGENTA clinical trial, which suggested a lack of significant impact on preventing cerebral palsy. Therefore, further evaluation of this topic through the design of new randomized clinical trials is crucial. These trials will provide valuable insights and help clarify the effectiveness of MgSO_4_ for neuroprotection and its potential role in preventing cerebral palsy.

The present review demonstrates several strengths across various aspects. Firstly, the search conducted was comprehensive, encompassing well-known databases such as Medline/PubMed, Cochrane database (CENTRAL), and Clinicaltrials.gov (as detailed in Supplementary Table 1). This extensive search strategy ensures a thorough examination of relevant literature. Additionally, the inclusion of data from the newly published MAGENTA RCT provides our research with a competitive advantage. Furthermore, while the most recent meta-analysis on this topic by Wolf et al. did not fully explore the independent effects of Magnesium sulfate dosage and trial follow-up duration on CP or mortality rate, our systematic review and meta-analysis conducted subgroup analyses based on these variables.

The main limitation of our study was the limited number of studies available for inclusion due to the inadequate number of RCTs conducted on the use of MgSO_4_ administration in preterm deliveries for the prevention of CP. Additionally, a notable limitation we encountered while investigating the potential association between treatment and favorable outcomes was the lack of research and evidence from low-income countries. The absence of data from these regions highlights the need for conducting clinical trials specifically among nations with limited resources.

## Conclusion

To sum up, our results can confirm the overall positive impact of administrating Magnesium Sulfate in likely-to-happen preterm deliveries for neuroprotection. Our research consisted of recent studies analyzing this subject and could be considered as an update to what we already knew. Nevertheless, considering the recently published MAGENTA randomized clinical trial, questioning the very presumption of Magnesium Sulfate efficacy as a measure to prevent neurodevelopment impairments from ever occurring, future research is undeniably needed to assess the matter further. But, for the time being, MgSO_4_ is still the best and most viable medication for neuroprotection.

### Electronic supplementary material

Below is the link to the electronic supplementary material.

Supplementary Material 1

## Data Availability

All data has been presented in the manuscript.

## References

[1] Wagura P, Wasunna A, Laving A, Wamalwa D. Ng’Ang’a P. Prevalence and factors associated with preterm birth at kenyatta national hospital. BMC Pregnancy Childbirth. 2018;18(1):107. 29673331 10.1186/s12884-018-1740-2 PMC5909235

[2] Cao G, Liu J, Liu M, Global. Regional, and National Incidence and Mortality of neonatal Preterm Birth, 1990–2019. JAMA Pediatr. 2022;176(8):787–96. 35639401 10.1001/jamapediatrics.2022.1622 PMC9157382

[3] Darmstadt GL, Al Jaifi NH, Arif S, Bahl R, Blennow M, Cavallera V et al. New World Health Organization recommendations for care of preterm or low birth weight infants: health policy. EClinicalMedicine. 2023;63. 10.1016/j.eclinm.2023.102155 PMC10518507 37753445

[4] Ream MA, Lehwald L. Neurologic consequences of Preterm Birth. Curr Neurol Neurosci Rep. 2018;18(8):48. 29907917 10.1007/s11910-018-0862-2

[5] Chang E. Preterm birth and the role of neuroprotection. BMJ: Br Med J. 2015;350:g6661. 25646630 10.1136/bmj.g6661

[6] Bachnas MA, Akbar MIA, Dachlan EG, Dekker G. The role of magnesium sulfate (MgSO4) in fetal neuroprotection. J Maternal-Fetal Neonatal Med. 2021;34(6):966–78. 10.1080/14767058.2019.1619688 31092073

[7] Gano D, Ho M-L, Partridge JC, Glass HC, Xu D, Barkovich AJ, et al. Antenatal exposure to Magnesium Sulfate is Associated with reduced cerebellar hemorrhage in Preterm newborns. J Pediatr. 2016;178:68–74. 27453378 10.1016/j.jpeds.2016.06.053 PMC5085851

[8] Page MJ, McKenzie JE, Bossuyt PM, Boutron I, Hoffmann TC, Mulrow CD, et al. The PRISMA 2020 statement: an updated guideline for reporting systematic reviews. BMJ. 2021;372:n71. 33782057 10.1136/bmj.n71 PMC8005924

[9] Wolf HT, Brok J, Henriksen TB, Greisen G, Salvig JD, Pryds O, et al. Antenatal magnesium sulphate for the prevention of cerebral palsy in infants born preterm: a double-blind, randomised, placebo-controlled, multi-centre trial. BJOG. 2020;127(10):1217–25. 32237024 10.1111/1471-0528.16239

[10] Rouse DJ, Hirtz DG, Thom E, Varner MW, Spong CY, Mercer BM, et al. A Randomized, Controlled Trial of Magnesium Sulfate for the Prevention of Cerebral Palsy. N Engl J Med. 2008;359(9):895–905. 18753646 10.1056/NEJMoa0801187 PMC2803083

[11] Luu TM, Ment LR, Schneider KC, Katz KH, Allan WC, Vohr BR. Lasting effects of preterm birth and neonatal brain hemorrhage at 12 years of age. Pediatrics. 2009;123(3):1037–44. 19255037 10.1542/peds.2008-1162 PMC2651566

[12] Burguet A, Monnet E, Roth P, Hirn F, Vouaillat C, Lecourt-Ducret M, et al. [Neurodevelopmental outcome of premature infants born at less than 33 weeks of gestational age and not cerebral palsy at the age of 5 years]. Arch Pediatr. 2000;7(4):357–68. 10793922 10.1016/S0929-693X(00)88830-2

[13] Moreira RS, Magalhães LC, Alves CR. Effect of preterm birth on motor development, behavior, and school performance of school-age children: a systematic review. J Pediatr (Rio J). 2014;90(2):119–34. 24370176 10.1016/j.jped.2013.05.010

[14] González J, Vilella M, Ruiz S, Iglesia I, Clavero-Adell M, Ayerza-Casas A et al. Impact of suspected Preterm Labor during pregnancy on Cardiometabolic Profile and Neurodevelopment during Childhood: a prospective cohort study protocol. Diagnostics (Basel). 2023;13(6). 10.3390/diagnostics13061101 PMC10047113 36980410

[15] Steer CM, Petrie RH. A comparison of magnesium sulfate and alcohol for the prevention of premature labor. Am J Obstet Gynecol. 1977;129(1):1–4. 331952 10.1016/0002-9378(77)90808-0

[16] Conde-Agudelo A, Romero R. Antenatal magnesium sulfate for the prevention of cerebral palsy in preterm infants less than 34 weeks’ gestation: a systematic review and metaanalysis. Am J Obstet Gynecol. 2009;200(6):595–609. 19482113 10.1016/j.ajog.2009.04.005 PMC3459676

[17] Crowther CA, Ashwood P, Middleton PF, McPhee A, Tran T, Harding JE. Prenatal intravenous magnesium at 30–34 weeks’ Gestation and neurodevelopmental outcomes in offspring: the MAGENTA Randomized Clinical Trial. JAMA. 2023;330(7):603–14. 37581672 10.1001/jama.2023.12357 PMC10427942

[18] Wolf HT, Huusom LD, Henriksen TB, Hegaard HK, Brok J, Pinborg A. Magnesium sulphate for fetal neuroprotection at imminent risk for preterm delivery: a systematic review with meta-analysis and trial sequential analysis. BJOG. 2020;127(10):1180–8. 32237069 10.1111/1471-0528.16238

[19] Crowther CA, Middleton PF, Voysey M, Askie L, Duley L, Pryde PG, et al. Assessing the neuroprotective benefits for babies of antenatal magnesium sulphate: an individual participant data meta-analysis. PLoS Med. 2017;14(10):e1002398. 28976987 10.1371/journal.pmed.1002398 PMC5627896

[20] Jayaram PM, Mohan MK, Farid I, Lindow S. Antenatal magnesium sulfate for fetal neuroprotection: a critical appraisal and systematic review of clinical practice guidelines. J Perinat Med. 2019;47(3):262–9. 30352042 10.1515/jpm-2018-0174

[21] Magee LA, De Silva DA, Sawchuck D, Synnes A, von Dadelszen P. 376-Magnesium Sulphate for fetal neuroprotection. J Obstet Gynecol Can. 2019;41(4):505–22. 30879485 10.1016/j.jogc.2018.09.018

[22] Pearson S, Loubser L, Nguyen K, Curry A. Should magnesium sulfate be given for neuroprotection for preterm labor between 32 and 34 weeks’ gestation? Evidence-Based Pract. 2023;26(4):26–7. 10.1097/EBP.0000000000001813

[23] Nguyen TMN, Crowther CA, Wilkinson D, Bain E. Magnesium sulphate for women at term for neuroprotection of the fetus. Cochrane Database Syst Reviews. 2013(2). 10.1002/14651858.CD009395.pub2 PMC11683453 23450601

[24] Crowther CA, Middleton PF, Voysey M, Askie LM, Duley L, Pryde PG et al. Assessing the neuroprotective benefits for babies of antenatal magnesium sulphate: an individual participant data meta-analysis. PLoS Med. 2017;14. 10.1371/journal.pmed.1002398 PMC5627896 28976987

