# Supplementary material for: Magnesium sulfate for fetal neuroprotection in preterm pregnancy: a meta-analysis of randomized controlled trials
Source: BMC Pregnancy Childbirth. 2024 Aug 1;24:519. doi: 10.1186/s12884-024-06703-9 (PMC11295595; doi:10.1186/s12884-024-06703-9)

# Supplementary Material

**Magnesium sulfate for fetal neuroprotection in preterm pregnancy: an updated meta-analysis of randomized controlled trials.**

**Supplementary Table 1.** Search strategies for online databases

**Supplementary Figure 1.** Results of subgroup analysis.

This supplemental material has been provided by the authors to give readers additional information about their work.

**Supplementary Table 1.** Search strategies for online databases

| **MEDLINE (via PubMed)** | ("Magnesium sulphate "[MeSH Terms] OR " MgSO4 "[Title/Abstract]) AND ("preterm delivery"[MeSH Terms] OR "preterm birth"[Title/Abstract] AND ("neuroprotection "[MeSH Terms] OR " cerebral palsy "[Title/Abstract] OR “death"[Title/Abstract] |
| --- | --- |
| **Cochrane Central Register of Controlled Trials (CENTRAL, via The Cochrane Library)** | ((Magnesium sulphate) AND ("cerebral palsy" OR death OR neuroprotection)) AND ("randomized controlled trial" OR RCT) AND (preterm delivery) |

**Supplementary Figure 1.** Results of subgroup analysis for fetal neurological impairment a) dose; and b) follow-up.

a)


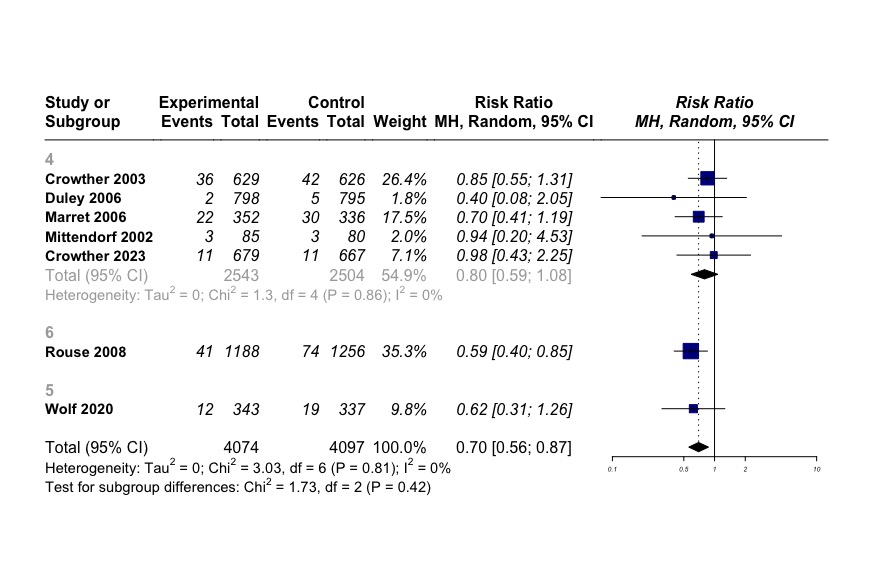


b)
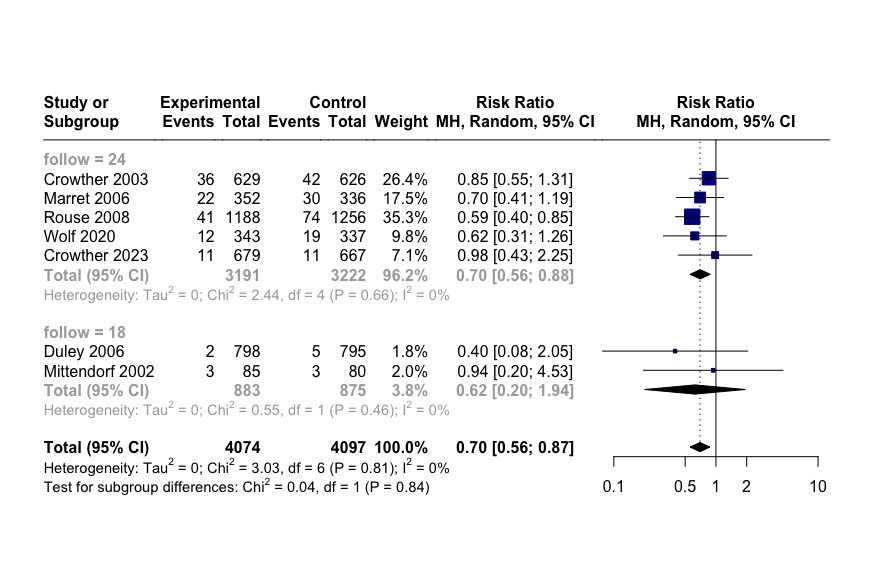


**Supplementary Figure 3.** Results of subgroup analysis for mortality a) dose; and b) follow-up.

a)
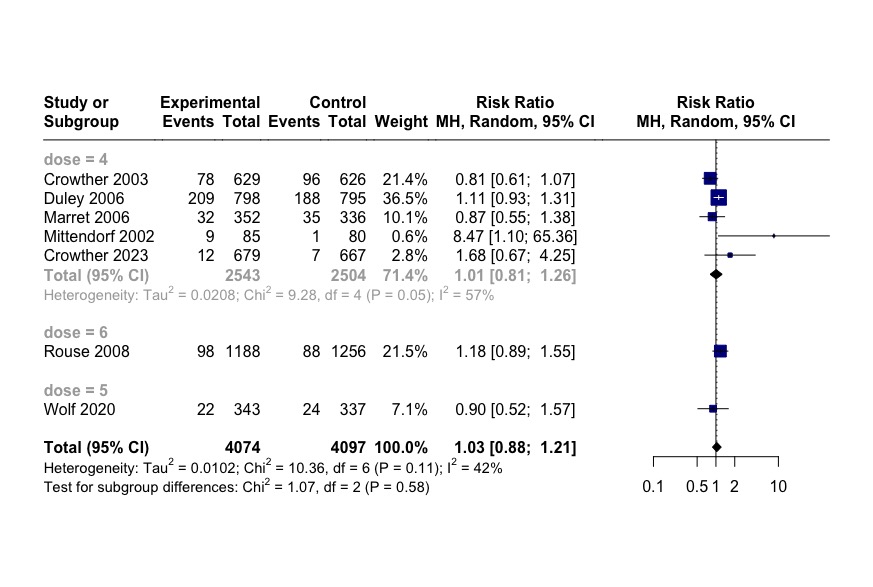


b)


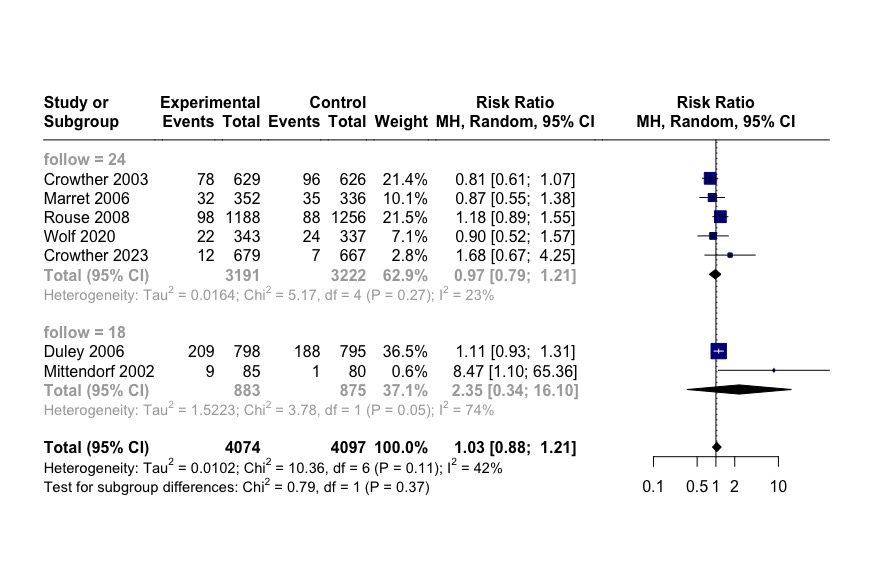

Supplement: Supplementary file 1 — Supplementary Material 1 [file 12884_2024_6703_MOESM1_ESM.docx]
